# Clinical Characteristics and Treatment Outcomes of COVID-19 Patients at Eka Kotebe General Hospital, Addis Ababa, Ethiopia

**DOI:** 10.4269/ajtmh.21-1270

**Published:** 2022-07-05

**Authors:** Dawit Kebede Huluka, Eyob Kebede Etissa, Sebrina Ahmed, Hiluf Abate Abule, Nebiyu Getachew, Sisay Abera, Abebaw Bekele Seyoum, Hiruy Araya, Tsegaye Gebreyes Hundie, Bethlehem Tadesse Anteneh, Getachew Demoz Gebremedhin, Yonas Gebregziabher, Rediet Yitagesu Tefera, Addisu Birhanu Tereda, Yohannes Feleke, Yonathan Abebe, Tewodros Haile Gebremariam, Hanan Yusuf Ahmed, Wondwossen Amogne, Deborah A. Haisch, Charles B. Sherman, Neil W. Schluger

**Affiliations:** ^1^ College of Health Sciences, Addis Ababa University, Addis Ababa, Ethiopia;; ^2^ East African Training Initiative, Addis Ababa, Ethiopia;; ^3^ Eka Kotebe Hospital, Addis Ababa, Ethiopia;; ^4^ Weill Cornell Medical College, New York, New York;; ^5^ Warren Alpert School of Medicine, Brown University, Providence, Rhode Island;; ^6^ Westchester Medical Center, New York Medical College, New York, New York

## Abstract

Data from much of Africa are still scarce on the clinical characteristics, outcomes of treatment, and factors associated with disease severity and mortality of COVID-19. A cross-sectional study was conducted at Eka Kotebe General Hospital, Ethiopia’s first COVID-19 treatment center. All consecutive symptomatic SARS CoV-2 RT-PCR positive individuals, aged 18 and older, admitted to the hospital between March 13 and September 16, 2020, were included. Of the total 463 cases, 319 (68.9%) were male. The median age was 45 years (interquartile range 32–62). The most common three symptoms were cough (69%), shortness of breath (SOB; 44%), and fatigue (37%). Hypertension was the most prevalent comorbidity, followed by diabetes mellitus. The age groups 40 to 59 and ≥ 60 were more likely to have severe disease compared with those < 40 years of age (adjusted odds ratio [aOR] = 3.45, 95% confidence interval [CI]: 1.88–6.31 and aOR = 3.46, 95% CI: 1.91–6.90, respectively). Other factors associated with disease severity included the presence of any malignancy (aOR = 4.64, 95% CI: 1.32–16.33) and SOB (aOR = 3.83, 95% CI: 2.35–6.25). The age group ≥ 60 was significantly associated with greater in-hospital mortality compared with those < 40 years. In addition, the presence of any malignancy, SOB, and vomiting were associated with higher odds of mortality. In Ethiopia, most COVID-19 patients were male and presented with cough, SOB, and fatigue. Older age, any malignancy, and SOB were associated with disease severity; these factors, in addition to vomiting, also predicted mortality.

## INTRODUCTION

As of December 2, 2021, there were more than 261 million COVID-19 cases and 5.2 million verified COVID-19 deaths worldwide. In Africa, there were approximately 8.8 million cases and 224,000 deaths. Most who contract the virus are asymptomatic, but the majority of symptomatic patients will have mild to moderate respiratory disease. On the other hand, older individuals and those with comorbidities can become severely ill and require medical attention. However, people of any age can become extremely ill or die of the disease.^1^^–^^3^

In the initial WHO-China Joint Mission on Coronavirus Disease 2019 and according to a comprehensive overview and meta-analysis, the most common disease symptoms are fever, cough, fatigue, sputum, dyspnea, myalgia, chest tightness/pain, sore throat, headache, diarrhea, nasal congestion/rhinorrhea, nausea/vomiting, abdominal discomfort, and hemoptysis. In most individuals, COVID-19-related comorbidities include hypertension (HTN), diabetes mellitus (DM), and cardiovascular disease. Some researchers have reported additional comorbidities of endocrine disorders, gastrointestinal ailments, chronic liver disease, and chronic obstructive pulmonary disease (COPD).^4^^–^^8^

Older age; comorbidities such as DM, HTN, cardiovascular illness, and chronic respiratory disease, and the symptom of shortness of breath (SOB) have been identified as predictors of disease severity and mortality.^9^^–^^20^ Others have reported risk factors for disease severity to be male gender, low oxygen saturation (SpO_2_), two or more comorbidities, malignancy, chronic kidney disease (CKD), human immunodeficiency virus (HIV), obesity, smoking, cough, fever, and fatigue.^9^^,^^10^^,^^17^^,^^18^^,^^20^

COVID-19 mortality has been associated with decreased SpO_2_, CKD, malignancy, HIV/AIDS, and fever.^9^^,^^15^^,^^21^ Others report male gender, greater sequential organ failure assessment, and previous and current tuberculosis.^15^^,^^21^^,^^22^

As of this writing, it has been 1.5 years since the first case was reported in Ethiopia. As of December 2, 2021, 371,272 cases and 6,771 deaths had been reported in the nation.^23^^,^^24^ Only a few studies have been published from Ethiopia on the clinical features, illness severity, treatment, and outcomes. This study aimed to describe the clinical manifestations, treatment, outcomes, and factors related to severity and mortality at Eka Kotebe Hospital, Ethiopia's pioneer COVID-19 treatment facility.

## METHODS

### Study design and setting.

This cross-sectional retrospective study was undertaken in Eka Kotebe General Hospital, the first COVID-19 treatment center in Addis Ababa. It was initially established as an extension of the Amanuel General Hospital until April 2020 when it became a stand-alone federal hospital. It has a bed capacity of approximately 400, with 40 beds dedicated to intensive care services, 16 of which are for patients requiring mechanical ventilation (such as critically sick COVID-19 patients). Patients of all levels of severity (mild to critical COVID-19) were admitted to the hospital. It is staffed by more than 130 nurses, 90 general practitioners, three anesthesiologists, three emergency physicians, two internists, one pulmonary and critical care subspecialist, two obstetrics and gynecology physicians, two surgeons, three psychiatrists, two radiologists, and two pediatricians. Nine of these senior physicians are academic staff at the College of Health Sciences, Addis Ababa University, and they have been working in the hospital since April 2020.

### Study period.

The study took place from March 13, 2020, through September 16, 2020.

### Study population.

All consecutive symptomatic SARS CoV-2 Reverse Transcriptase Polymerase Chain Reaction (RT-PCR) positive were included.

### Inclusion criteria.

Regardless of data completeness, all COVID-19 patients aged 18 and older were included. Only those who were symptomatic and those with positive RT-PCR on admission or who turned positive after admission were included.

### Exclusion criteria.

Asymptomatic cases and those with SARS CoV-2 RT-PCR–negative test results who were admitted to the hospital early in the pandemic when it was serving as both an isolation and a quarantine center.

### Sample size.

All cases meeting the inclusion criteria during the study period were included.

### Operational definitions.

- COVID-19 patient: An individual who had a positive RT-PCR irrespective of symptoms.
- Asymptomatic COVID-19: Individuals who had a positive RT-PCR with no symptoms suggestive of COVID-19.
- Symptomatic COVID-19: Individuals who had a positive RT-PCR with one or more of the symptoms suggestive of COVID-19 including fever, cough, headache, myalgia, arthralgia, loss of smell/taste sensation, vomiting, and diarrhea.
- Mild disease: A symptomatic COVID-19 case with no radiologic finding who did not require oxygen and had a normal hemodynamic status.
- Moderate disease: A symptomatic COVID-19 case with radiographic evidence of infiltrates or pneumonia and SpO_2_ ≥ 90%.
- Severe disease: A COVID-19 case with SpO_2_ < 90% irrespective of symptoms or radiographic findings.
- Critical COVID: A COVID-19 case requiring mechanical ventilation or hemodynamic support. This includes patients with acute respiratory distress syndrome, acute renal failure, and septic shock.
- Disease severity: Nonsevere COVID-19 (mild to moderate cases) and severe COVID-19 (severe or critical cases).
- Chronic lung diseases included preexisting COPD and bronchial asthma.

### Data collection and quality assurance.

A structured questionnaire was used to collect data on demographics, clinical manifestations, comorbidities, laboratory values, inpatient medications, treatments (including invasive mechanical ventilation and kidney replacement therapy), and outcomes (including length of stay, discharge, readmission, and mortality) of the study subjects. Trained physician data clerks collected data from the chart. The questionnaire was tested, and revisions were made before data collection started.

### Data analysis.

The collected data were coded, entered into CSPro software, and exported to SPSS version 26 for analysis. Categorical variables were presented using frequency and percentages, whereas continuous variables were reported as medians with interquartile ranges (IQRs). For categorical variables, the chi-square or Fisher exact test for expected frequency < 5 in univariate analysis was used to make a comparison between groups. An independent *t* test for continuous variables was performed to compare the means of two independent groups for normally distributed and the Mann-Whitney *U* test for nonnormally distributed numeric data. To determine the predictor of disease severity (nonsevere versus severe) and COVID-19 outcome (alive or dead during hospital stay), a binary logistic regression model was used independently. In the univariate analysis, variables with *P* < 0.1 were used to identify potential significant factors for the final models. A binary logistic regression model was well fitted to identify predictor variables Hosmer and Lemeshow goodness of fit test *P* = 0.126 and *P* = 0.055 for disease severity and mortality outcome respectively. Adjusted odds ratio (aOR) with a 95% confidence interval (CI) and *P* value < 0.05 was used as statistically significant.

### Source of funding and ethical consideration.

This study was supported by the East African Training Initiative. Ethical clearance was obtained from the Institutional Review Board of Eka Kotebe General Hospital (ref. no. Yek/150/5/9). All data managers and collectors received the same training on maintaining confidentiality.

## RESULTS

A total of 463 laboratory-confirmed symptomatic COVID-19 patients met the inclusion criteria; 319 (68.9%) were male. The median age was 45 years (IQR: 32–62); 38.2% (*n* = 177) of subjects were younger than 40 years. Cough (*n* = 313, 68.6%), SOB (*n* = 204, 44.1%), fatigue/malaise (*n* = 171, 36.9%) fever (*n* = 162, 35.0%), and headaches (*n* = 131, 28.3%) were the most common symptoms. Comorbidities were present in 189 (40.8%) of the participants. HTN (*N* = 112, 24.2%), DM (*n* = 96, 20.7%), and chronic cardiac diseases (*n* = 27, 5.8%) were the most prevalent comorbidities (Table 1).

**Table 1:** Baseline characteristics, comorbidities, and admission symptoms of respondents in Eka Kotebe Hospital

Variables	*n* (%)
Age (median: 45, IQR: 32–62)	
< 40	177 (38.2)
40–59	147 (31.7)
≥ 60	139 (30.0)
Sex	
Male	319 (68.9)
Female	144 (31.1)
Comorbidities and symptoms	
Comorbidity	
Yes	189 (40.8)
No	274 (59.2)
Hypertension	
Yes	112 (24.2)
No	351 (75.8)
Type 2 diabetes mellitus	
Yes	96 (20.7)
No	367 (79.3)
Chronic cardiac disease	
Yes	27 (5.8)
No	436 (94.2)
Chronic lung disease	
Yes	21 (4.5)
No	442 (95.5)
Malignancy	
Yes	19 (4.1)
No	444 (95.9)
HIV/AIDS	
Yes	11 (2.4)
No	452 (97.6)
Chronic kidney disease	
Yes	8 (1.7)
No	455 (98.3)
Obesity	
Yes	6 (1.4)
No	413 (98.6)
Chronic liver disease	
Yes	2 (0.4)
No	461 (99.6)
Cough	
Yes	313 (68.6)
No	150 (32.4)
Shortness of breath	
Yes	204 (44.1)
No	259 (55.9)
Fatigue/malaise	
Yes	171 (36.9)
No	292 (63.1)
Fever	
Yes	162 (35.0)
No	301 (65.0)
Headache	
Yes	131 (28.3)
No	332 (71.7)
Myalgia	
Yes	110 (23.8)
No	353 (76.2)
Arthralgia	
Yes	109 (23.5)
No	354 (76.5)
Loss of appetite	
Yes	89 (19.2)
No	374 (80.8)
Sore throat	
Yes	65 (14.0)
No	398 (86.0)
Chills	
Yes	40 (8.6)
No	423 (91.4)
Vomiting	
Yes	31 (6.7)
No	432 (93.3)
Abdominal pain	
Yes	29 (6.3)
No	434 (93.7)
New loss of smell	
Yes	26 (5.6)
No	434 (94.4)
New loss of taste	
Yes	22 (4.8)
No	437 (95.2)
Diarrhea	
Yes	19 (4.1)
No	441 (95.9)
Nausea	
Yes	17 (3.7)
No	444 (96.3)
Rhinorrhea	
Yes	17 (3.7)
No	446 (96.3)

IQR = interquartile range.

An absolute lymphocyte count (ALC) < 1,000/mm^3^ was seen in 140 (34.7%) subjects, aspartate transaminase (AST) > 37 u/L (reference range up to 37) in 136 (38.2%), and alanine transaminase (ALT) > 63 u/L (up to 63 reference range) in 68 (18.9%). Antibiotics (*n* = 290, 62.6%), corticosteroid (*n* = 109, 23.5%), chloroquine (*n* = 38, 8.2%), prophylactic anticoagulation (*n* = 201, 43.4%), full-dose anticoagulation (*n* = 60, 13.3%), and vasopressor support (*n* = 32, 6.9%) were administered to study subjects. Oxygen therapy was provided to 231 (49.9%), prone ventilation (either awake or with mechanical ventilation) to 149 (32.2%), invasive mechanical ventilation to 37 (8.0%), and noninvasive positive pressure ventilation to 26 (5.6%) patients. Tracheostomy was done for eight (1.7%) study participants. One hundred seventy-eight patients (38.4%) had severe and critical disease, and the remaining 285 (61.6%) had mild or moderate severity. The median duration of hospital stay was 15 days (IQR: 14–21). Case fatality was 11.4% (53 of 463) (Table 2).

**Table 2:** Laboratory findings and clinical management of study participants in Eka Kotebe Hospital

Variables		*n* (%)
Oxygen therapy	Yes	231 (49.9)
	No	232 (50.1)
Noninvasive positive pressure ventilation	Yes	26 (5.6)
	No	437 (94.4)
Invasive ventilation	Yes	37 (8.0)
	No	426 (92.0)
Prone ventilation	Yes	149 (32.2)
	No	314 (67.8)
Tracheostomy inserted	Yes	8 (1.7)
	No	455 (98.3)
Chloroquine administered	Yes	38 (8.2)
	No	425 (91.8)
Antibiotics	Yes	290 (62.6)
	No	173 (37.4)
Steroids	Yes	109 (23.5)
	No	354 (76.5)
Prophylactic anticoagulant	Yes	201 (43.4)
	No	262 (56.6)
Full dose anticoagulant	Yes	60 (13.0)
	No	403 (87.0)
Vasopressor required	Yes	32 (6.9)
	No	431 (93.1)
ALC (*N* = 404)	< 1,000	140 (34.7)
	≥ 1,000	264 (65.3)
AST (*N* = 356)	< 37	220 (61.8)
	≥ 37	136 (38.2)
ALT (*N* = 360)	≤ 63	292 (81.1)
	> 63	68 (18.9)
Length of stay (median, IQR) (15, 14–21)		
Length of stay (days)	≤ 15	255 (55.1)
	> 15	208 (44.9)

ALC = absolute lymphocyte count; ALT = alanine transaminase; AST = aspartate transaminase; IQR = interquartile range.

A chi-square test result revealed a statistically significant difference in disease severity across patient groups based on age, gender, presence of any comorbidities, HTN, DM, chronic cardiac disease, chronic lung disease, malignancy, CKD, cough, SOB, fatigue/malaise, headache, and new loss of smell sensation (*P* < 0.05). A statistically significant proportion of patients aged ≥ 60 years had severe disease (45.0% versus 20.7%, *P* < 0.001) compared with nonsevere disease, whereas a statistically significant proportion of patients aged younger than 40 years had nonsevere disease (53.3% versus 14.0%, *P* < 0.001) compared with severe disease. A significantly higher proportion of patients having any comorbidity or HTN, DM, chronic cardiac disease, chronic lung disease, malignancy, CKD, cough, SOB, and fatigue/malaise had severe disease (Table 3).

**Table 3:** Demographic, comorbidity, and symptom characteristics; comparison based on disease severity and factors associated

Characteristics		All patients	Severity		*P* value	aOR (95% CI)	*P* value	aOR (95% CI)	*P* value
			Nonsevere (mild and moderate) (*n* = 285)	Severe (severe and critical) (*n* = 178)					
Age in years (median, IQR)		45 (32–62)	38 (29–54)	55 (45–67)	0.001
Age	< 40	177 (38.2)	152 (53.3)	25 (14.0)	0.001	1		1	
	40–59	147 (31.7)	74 (26.0)	73 (41.0)		5.998 (3.522–10.215)	0.001	3.445 (1.882–6.307)	0.000*
	≥ 60	139 (30.0)	59 (20.7)	80 (45.0)		8.244 (4.802–14.153)	0.001	3.627 (1.906–6.901)	0.000*
Sex n (%)	Male	319 (68.9)	206 (72.3)	113 (63.5)	0.047	0.667 (0.447–0.995)	0.047	0.841 (0.507–1.395)	0.503
	Female	144 (31.1)	79 (27.7)	65 (36.5)		1		1	
Comorbidity		189 (40.8)	77 (27.0)	112 (62.9)	0.001	4.584 (3.069–6.846)	0.001	1.233 (0.552–2.754)	0.610
Hypertension		112 (24.2)	41 (14.4)	71 (39.9)	0.000	3.949 (2.527–6.172)	0.001	1.664 (0.795–3.483)	0.177
Type 2 diabetes mellitus		96 (20.7)	37 (13.0)	59 (33.1)	0.001	3.323 (2.086–5.293)	0.001	1.559 (0.792–3.070)	0.199
Chronic cardiac disease		27 (5.8)	10 (3.5)	17 (9.6)	0.007	0.344 (0.154–0.770)	0.009	0.950 (0.358–2.519)	0.917
Chronic lung disease		21 (4.5)	7 (2.5)	14 (7.9)	0.007	3.390 (1.341–8.572)	0.010	1.822 (0.593–5.595)	0.295
Malignancy		19 (4.1)	7 (2.5)	12 (6.7)	0.024	2.871 (1.108–7.436)	0.030	4.641 (1.319–16.337)	0.017*
HIV/AIDS		11 (2.4)	6 (2.1)	5 (2.8)	0.756				
Chronic kidney disease		8 (1.7)	1 (0.4)	7 (3.9)	0.006	11.626 (1.418–95.305)	0.022	3.084 (0.340–27.990)	0.317
CLD		2 (0.4)	1 (0.4)	1 (0.6)	0.736				
Cough		313 (67.6)	179 (62.8)	134 (75.3)	0.005	1.803 (1.189–2.736)	0.006	1.122 (0.664–1.896)	0.666
Shortness of breath		204 (44.1)	82 (28.8)	122 (68.5)	0.001	5.393 (3.589–8.104)	0.001	3.831 (2.347–6.252)	0.000*
Fatigue/malaise		171 (36.9)	88 (30.9)	83 (46.6)	0.001	1.956 (1.328–2.880)	0.001	1.456 (0.883–2.401)	0.141
Fever		162 (35.0)	93 (32.6)	69 (38.8)	0.178				
Headache		131 (28.3)	95 (33.3)	36 (20.2)	0.002	0.507 (0.326–0.788)	0.003	0.630 (0.367–1.084)	0.095
Myalgia		110 (23.8)	63 (22.1)	47 (26.4)	0.290				
Arthralgia		109 (23.5)	63 (22.1)	46 (25.8)	0.356				
Loss of appetite		89 (19.2)	48 (16.8)	41 (23.0)	0.100				
Sore throat		65 (14.0)	45 (15.8)	20 (11.2)	0.170				
Chill		40 (8.6)	20 (7.0)	20 (11.2)	0.119				
Vomiting		31 (6.7)	16 (5.6)	15 (8.4)	0.239				
Abdominal pain		29 (6.3)	17 (6.0)	12 (6.7)	0.737				
New loss of smell		26 (5.6)	21 (7.4)	5 (2.8)	0.038	0.363 (0.134–0.982)	0.046	0.497 (0.146–1.699)	0.265
New loss of taste		22 (4.8)	17 (6.0)	5 (2.8)	0.120				
Diarrhea		19 (4.1)	14 (4.9)	5 (2.8)	0.267				
Nausea		17 (3.7)	13 (4.6)	4 (2.2)	0.198				
Runny nose (rhinorrhea)		17 (3.7)	13 (4.6)	4 (2.2)	0.198				

aOR = adjusted odds ratio; CI = confidence interval; CLD = chronic liver disease; IQR = interquartile range.

Age, malignancy, and SOB were significantly associated with COVID-19 severity in the multivariable binary logistic regression. The odds of having severe disease compared with nonsevere disease are 3.4 and 3.6 times greater in the 40 to 59 and 60 and older age groups than for patients younger than 40 years (aOR = 3.44, 95% CI: 1.88–6.31, *P* < 0.0001) and (aOR = 3.63, 95% CI: 1.91–6.90, *P* < 0.0001), respectively. The odds of having severe COVID-19 were 4.6 times higher in patients with the presence of malignancy (aOR = 4.64, 95% CI: 1.32–16.33, *P* = 0.017). The presence of SOB also increased the odds of having severe disease (aOR = 3.83, 95% CI: 2.35, 6.25, *P* < 0.0001) (Table 3).

The median age in those who died was older than in those who survived (61 versus 43 years, *P* < 0.0001), and fewer patients died in the age group below 40 than above 60 years (13.2 versus 60.4%, *P* < 0.0001). On univariate analysis HTN, DM, the presence of any comorbidity, malignancy, chronic liver disease (CLD), SOB, loss of appetite, vomiting, AST ≥ 37 u/L were significantly associated with in-hospital mortality whereas headache, loss of appetite and ALC count > 1,000/mm^3^ were found to decrease mortality (Table 4).

**Table 4:** Demographic, comorbidity, and symptom characteristics; comparison based on disease outcome and factors associated

Characteristics		All patients	Outcome		*P* value	aOR (95% CI)	*P* value	aOR (95% CI)	*P* value
			Death (*n *= 53)	Alive (*n* = 410)					
Age in years (median, IQR)		45 (32–62)	61 (47–70)	43 (31–60)	< 0.0001
Age	< 40	177 (38.2)	7 (13.2)	170 (41.5)	< 0.0001	1		**1**	
	40–59	147 (31.7)	14 (26.4)	133 (32.4)		2.556 (1.003–6.513)	0.049	1.463 (0.519,4.121)	0.471
	≥ 60	139 (30.0)	32 (60.4)	107 (26.1)		7.263 (3.096–17.041)	0.000	**3.935 (1.437–10.779)**	0.008*
Sex, *n* (%)	Male	319 (68.9)	31 (58.5)	288 (70.2)	0.082	0.597 (0.332–1.072)	0.084	0.955 (0.484–1.885)	0.894
	Female	144 (31.1)	22 (41.5)	122 (29.8)		1		1	
Comorbidity		189 (40.8)	36 (67.9)	153 (37.3)	0.000	3.557 (1.932–6.550)	0.000	0.905 (0.315–2.606)	0.854
Hypertension		112 (24.2)	23 (43.4)	89 (21.7)	0.001	2.765 (1.530–4.997)	0.001	1.198 (0.472–3.043)	0.704
Type 2 diabetes mellitus		96 (20.7)	19 (35.8)	77 (18.8)	0.004	2.417 (1.308–4.464)	0.005	1.880 (0.816–4.331)	0.138
Chronic cardiac disease		27 (5.8)	5 (9.4)	22 (5.4)	0.218				
Malignancy		19 (4.1)	7 (13.2)	12 (2.9)	0.003	5.047 (1.893–13.459)	0.001	**9.028 (2.463–33.092)**	0.001*
HIV/AIDS		11 (2.4)	1 (1.9)	10 (2.4)	1.000				
Chronic kidney disease		8 (1.7)	3 (5.7)	5 (1.2)	0.052	4.860 (1.127–20.952)	0.034	1.659 (0.317–8.678)	0.549
Chronic lung disease		21 (4.5)	5 (9.4)	16 (3.9)	0.069	2.565 (0.899–7.315)	0.078	1.838 (0.539–6.266)	0.331
CLD		2 (0.4)	2 (2.8)	0 (0.0)	0.013	1.299E+10 (0.000)	0.999		
Cough		313 (67.6)	42 (79.2)	271 (66.1)	0.054	1.958 (0.978–3.923)	0.058	1.411 (0.642–3.103)	0.391
Shortness of breath		204 (44.1)	37 (69.8)	167 (40.7)	0.000	3.365 (1.813–6.247)	0.000	**2.336 (1.148–4.752)**	0.019*
Fatigue/malaise		171 (36.9)	24 (45.3)	147 (35.9)	0.181				
Fever		162 (35.0)	21 (39.6)	141 (34.4)	0.452				
Headache		131 (28.3)	8 (15.1)	123 (30.0)	0.023	0.415 (0.190–0.906)	0.027	0.542 (0.230–1.279)	0.162
Myalgia		110 (23.8)	12 (22.6)	98 (23.9)	0.839				
Arthralgia		109 (23.5)	10 (18.9)	99 (24.10)	0.394				
Loss of appetite		89 (19.2)	16 (30.2)	73 (17.8)	0.031	1.996 (1.054–3.781)	0.034	1.329 (0.631–2.797)	0.454
Sore throat		65 (14.0)	3 (5.7)	62 (15.1)	0.062	0.337 (0.102–1.114)	0.074	0.503 (0.141–1.794)	0.290
Chill		40 (8.6)	4 (7.5)	36 (8.8)	1.000				
Vomiting		31 (6.7)	9 (17.0)	22 (5.4)	0.005	3.607 (1.564–8.322)	0.003	**3.049 (1.126–8.255)**	0.028*
Abdominal pain		29 (6.3)	5 (9.4)	24 (5.9)	0.359				
New loss of smell		26 (5.6)	1 (1.9)	25 (6.1)	0.341				
New loss of test		22 (4.8)	1 (1.9)	21 (5.1)	0.494				
Diarrhea		19 (4.1)	1 (1.9)	18 (4.4)	0.711				
Nausea		17 (3.7)	3 (5.7)	14 (3.4)	0.428				
Runny nose (rhinorrhea)		17 (3.7)	1 (1.9)	16 (3.9)	0.707				
ALC (*n* = 404)	< 1,000	140 (34.7)	34 (66.7)	106 (30.0)	0.000				
	≥ 1,000	264 (65.3)	17 (33.3)	247 (70.0)					
AST (*n* = 356)	< 37	220 (61.8)	13 (28.9)	207 (66.6)	0.000				
	≥ 37	136 (38.2)	32 (71.1)	104 (33.4)					
ALT (*n* = 360)	≤ 63	292 (81.1)	34 (73.9)	258 (82.2)	0.182				
	> 63	68 (18.9)	12 (26.1)	56 (17.8)					

ALC = absolute lymphocyte count; ALT = alanine transaminase; AST = aspartate transaminase; IQR = interquartile range.

Patients aged 60 and older had a 3.9-fold increased risk of mortality compared with patients younger than 40 (aOR = 3.94, 95% CI: 1.44–10.78, *P* = 0.008). After adjusting for covariates, age, malignancy, SOB, and vomiting were significantly associated with mortality in the multivariable binary logistic regression. Patients with malignancy were 9 times (aOR = 9.03, 95% CI: 2.46–33.09, *P* < 0.001) more likely to die. Patients with SOB had a 2.3 times (aOR = 2.34, 95% CI: 1.15–4.75, *P* = 0.019) higher risk of mortality than those without. Patients with vomiting had a more than 3-fold higher odds of death compared with those who did not (aOR = 3.04, 95% CI: 1.13–8.26, *P* = 0.028) (Table 4).

## DISCUSSION

This study investigated the clinical manifestations, treatment, outcomes, and factors related to the severity and mortality of COVID 19 in patients admitted to a COVID-19 specialty hospital in Addis Ababa, Ethiopia, in the prevaccine era. In our treatment center, more than two-thirds of participants were male. The median age was 45 years with 30% of study participants ≥ 60 years of age. Cough, SOB, fatigue/malaise, fever, and headaches were the most common symptoms. HTN, DM, and chronic cardiac diseases were the most frequent comorbidities. Overall, age ≥ 60 years, malignancy, and SOB were found to be significant predictors of disease severity; these factors, in addition to vomiting, also predicted mortality.

The reported rate of bacterial superinfection has been variable ranging from 8% in earlier clinical studies to 32% from autopsy reports.^25^^,^^26^ A recent more objective study based on bronchoalveolar lavage samples within 48 hours of hospitalization revealed 21% of superinfection.^27^ However, in our study, three out of five patients received antibiotics. This practice was predicated on the universal recommendation of antibiotic use in moderate to critical disease conditions in the previous national guidelines.^28^^,^^29^

More than half of the patients received anticoagulants, prophylactic or therapeutic, in accordance with the observed benefit of these medications in COVID-19, particularly those with severe disease.^30^ Slightly less than a quarter of patients were given corticosteroids, in contrast to current evidence that steroids have a survival advantage in severe to critical COVID-19.^31^ This underuse of steroids was due, in part, to the discretion of the managing team before publication of the interim report of the RECOVERY trial.

HTN, DM, and chronic cardiac disease were the most prevalent comorbidities. This finding is consistent with results from previous Ethiopian reports^12^^,^^32^ and other studies done in Africa, China, Brazil, and the United States.^9^^,^^14^ In multivariable analyses, HTN and DM were not associated with disease severity. This is in contrast to most studies. Another local study by Abraha et al. also found no association between HTN and severity of disease. However, DM, was associated with disease severity.^32^ COVID-19 in-hospital mortality was not associated with HTN or DM in multivariable analyses, similar to previous reports from Ethiopia, Saudi Arabia, Brazil, the U.S.–Mexico border, and the United States.^9^^,^^14^ HTN did not also increase risk for death in the largest COVID registry from United Kingdom.^33^ Further, HIV/AIDS was not associated with disease severity or mortality in our study. This finding agrees with other Ethiopian published reports^32^^,^^34^^,^^35^ and those from other areas of Africa, Europe, China, and the United States.^9^^,^^15^^,^^21^

Age was significantly associated with disease severity and in-hospital mortality, which was similar to the findings of other Ethiopian, African, and international studies.^1^^,^^9^^–^^11^^,^^13^^,^^14^^,^^16^^,^^20^^,^^21^^,^^32^^,^^33^^,^^35^^–^^38^ Proposed explanations include the physiological aging process, particularly the increased prevalence of frailty, age-related decline in lung function, comorbidities, and a weakened immune system.^39^^,^^40^ More than half of our study participants were < 50 years of age. Our age distribution was similar to other treatment centers in Ethiopia^32^^,^^34^^,^^35^ and data from other sub-Saharan countries,^41^^–^^43^ but younger than study populations reported from Europe, North America, and China.^44^ This variation could be due to the generally younger population of the African continent and greater hospital admissions in the region early during the pandemic for those with mild COVID-19 disease.

Patients with malignancy had adjusted odds ratio (aOR) 4.6 times greater for severe disease and 9 times greater for mortality compared with nonsevere disease. These findings are consistent with those of another Ethiopian study by Hiluf et al. from Tigray.^32^ It might be because of weakened immunity from the malignancy itself or from the immunosuppressive drugs used to treat the condition. The presence of SOB was associated with more than 3-fold increased odds of severe disease compared with nonsevere disease, and the risk of death was 2.3 times higher. This is in accordance with previously published studies.^9^^,^^11^^–^^13^^,^^18^^,^^20^ It might be because SOB occurs late in the course, usually in the inflammatory stage of the disease when mortality is high. Vomiting was also significantly associated with an increased likelihood of mortality. This is consistent with a report from Iraq that showed a poor prognosis in those with concomitant respiratory symptoms.^45^ However, it is contrary to reports from the United States.^46^^–^^48^

There are several study limitations. The lack of comprehensive laboratory findings prohibited us from including them in the final model as possible predictors of disease outcome. The cross-sectional nature of the study design made it difficult to establish a cause-effect relationship between the various factors and disease severity or treatment outcome. Being a single-center and hospital-based study, the findings may not be generalizable.

In conclusion, in Ethiopia, most COVID-19 patients were male and presented with cough, SOB, and fatigue. Older age, any malignancy, and SOB were associated with disease severity; these factors, in addition to vomiting, also predicted mortality.

## Financial Disclosure

This study was supported by the East African Training Initiative.
